# Improved Accuracy and Quality of Information During Emergency Department Care Transitions

**DOI:** 10.5811/westjem.2016.12.30858

**Published:** 2017-02-27

**Authors:** Nnaemeka Okafor, Justin Mazzillo, Sara Miller, Kimberly A. Chambers, Samar Yusuf, Vanessa Garza-Miranda, Yashwant Chathampally

**Affiliations:** University of Texas, Houston Health Science Center, Department of Emergency Medicine, Houston, Texas

## Abstract

**Introduction:**

Suboptimal communication during emergency department (ED) care transitions has been shown to contribute to medical errors, sometimes resulting in patient injury and litigation. The study objective was to determine whether a standardized checkout process would decrease the number of relevant missed clinical items (MCI).

**Methods:**

In this prospective pre- and post-intervention study conducted in an urban academic ED, we collected data on omitted or inaccurately conveyed medical information before and after the initiation of a standardized checkout process. The intervention included group checkout in an optimal location, review of electronic medical records, case discussion and assigned roles. MCI were considered relevant if they resulted in a delay or alteration in disposition or treatment plan. The primary outcome was the change in the number of MCI. Secondary outcomes were duration of checkout and physician satisfaction with the intervention.

**Results:**

Pre-intervention, there were 94 relevant MCI during 164 care transitions. Post-intervention, there were 36 MCI in 157 transitions. The mean MCI per transition decreased by 58% from 0.57 (95% confidence interval [CI] [0.41, 0.73]) to 0.23 (95% CI [0.11–0.35]). Instituting the intervention did not lengthen checkout duration, which was 15 minutes (95% CI [13.81–16.19]) pre-intervention and 14 minutes (95% CI [12.99–15.01]) post-intervention. The majority of participants (73.4%) felt that the process decreased MCI, and 89.5% reported that the new process had a positive or neutral effect on their satisfaction with care transitions.

**Conclusion:**

The adoption of a standardized care transition process markedly decreased clinically relevant communication errors without lengthening checkout duration.

## INTRODUCTION

### Background

Patient care transitions, handoffs, or checkouts are defined as a transfer of information, professional responsibility, and the authority to act for a patient. Emergency department (ED) care transitions are generally regarded as one of the most error-prone events within the routine ED workflow.^1^ The ED is an inherently chaotic environment in which patient care transitions typically occur in an informal manner. Due to a lack of standardization, checkout process variation is ubiquitous in emergency medicine.^1^

### Importance

A Joint Commission statement on sentinel events found that 84% involved a breakdown in communication, usually between physicians, with 62% relating to continuum of care issues.^2^ In addition, one study found that 24% of liability claims against ED providers included care transitions as a contributing factor.^3^ Previous studies have described a subjective decrease in the handoff error rate and an increase in physician satisfaction with the use of standardized transfer of care processes. Despite these results, there has been little consensus regarding the most valuable components of standardization. Research is limited due to a lack of objective measurements of error reduction after the implementation of checklists or other standard processes. Multiple sources including the Joint Commission, American College of Emergency Physicians (ACEP), and a survey of emergency medicine (EM) residency directors have noted the need for improved standardization of patient checkout for a more complete transfer of information.

At our institution, the need to improve our ED checkout process was identified through quality assurance review of error incidents that revealed care transitions-related communication gaps as significant contributing factors. Observation of EM resident care transitions revealed that wide variation occurs, which has been reported at other institutions as well.^4^

Resident care transitions were highly dependent on individual physician preferences and the state of the department at shift change. Resident handoffs did not occur as a group or in a standard location, were often interrupted by the nursing staff, and did not routinely involve supervising faculty. Patient data were typically reviewed after, rather than during, the care transition.

### Goals of This Investigation

We hypothesized that initiating a standardized group checkout process that included review of pertinent patient data would decrease the amount of relevant clinical information that was either omitted or inaccurately transferred. Further, we hypothesized that this intervention would not lengthen the duration of ED care transitions and that it would improve physician satisfaction.

## METHODS

### Study Design

We used an interventional study design to evaluate the impact of a standardized process for EM resident care transitions. No protected health information was used in the study, which met criteria for exemption from review by the University of Texas at Houston Institutional Review Board.

The primary outcome of interest was the change in the number of missed clinical items (MCI) pre and post-intervention. MCI were defined as relevant clinical items that were omitted or inaccurately conveyed and which resulted in a delay or alteration in the patient’s disposition or treatment plan. Secondary outcomes of interest were the types of MCI, checkout duration, and provider satisfaction with the process change.

**Population Health Research Capsule**

What do we already know about this issue?

Turning over patients is a high-risk event that can lead to medical errors. 84% of sentinel events involved a breakdown in communication, and 25% of liability cases in EM included transfer of care.

What was the research question?

Whether a standardized checkout process, compared to an unstructured one, would decrease the number of relevant miscommunications, defined as delay or change in patient management or disposition. Missed or poorly communicated items were counted and categorized, giving an objective primary outcome measure.

What was the major finding of the study?

The multifactorial change in the sign-out process led to fewer missed or inaccurately conveyed items of information, without prolonging checkout, and most providers judged it superior to unstructured care transitions.

How does this improve population health?

Emergency department crowding is frequent, and emergency physicians turn over boarding patients to colleagues commonly. Miscommunication is a documented problem, especially with critical illness or multiple active problems. Communication errors were decreased with a standardized checkout process, lowering the risk of delays and suboptimal care.

A data form (Appendix 1) was used during the care transition to record the checkout start and end time and the number of patients signed out. The data form was also used during the course of the shift to document the number and type of MCI.

During the study’s two-month pre-intervention phase, ED care transitions were conducted in accordance with previous practice patterns.

Prior to the initiation of the intervention, residents and faculty received instructions about the standardized care transition process at the weekly educational conference and through presentations transmitted via email. Additionally, during the first few days of the intervention, providers received in-person guidance from study personnel.

### Study Setting and Population

We conducted the study between February and May 2012 in an urban tertiary care Level 1 trauma center with 60,000 annual ED patient visits and an EM residency program. The pre-intervention phase occurred during February and March. The post-intervention phase directly followed during April and May.

The study subjects were the EM residents and faculty who participated in care transitions during EM resident shift change, which occurred daily at 0700 and 1900 in two care areas. One area included primarily major and minor medical complaints. The second is designated primarily, but not exclusively, for major and minor traumatic chief complaints. The patient volume and resident staffing was approximately equal in the two areas. Resident staff included EM residents at post-graduate year (PGY) 1, 2, and 3. At times, there were also off-service residents rotating in the treatment areas. There were at least two residents per area during a shift. In general, residents signed out to residents of similar experience; however, at times staffing required patients to be transferred to a resident of different training and experience. For example, a PGY-2 might sign out to a PGY-3 or PG-1. Of note, there were never two PGY-1 residents stationed in either treatment area alone; there was always at least one EM PGY-2 or -3 per side. Each part of the ED had a dedicated faculty member to supervise the two to three residents in that section.

All care transitions between ED residents were included in the study. We excluded care transitions that did not contain resident-to-resident patient checkouts. Twice per day (at 1500 and 2300), ED faculty changed shift without an associated change of resident staffing, and those transitions were not studied. Once per week, advanced practice professionals (APP) worked while EM residents were at the educational conference. Coverage by APP resulted in two care transitions per week (at 0700 and 1300 on Thursdays) between residents and APP. We also excluded those from the intervention and study.

The rationale for excluding non-resident-to-resident care transitions was multifactorial. First, ED faculty and APP did not work principally at the study hospital during the study period. As a result, their sign-out process may have had more variation and their training on the intervention may not have been as comprehensive as the residents’ instruction was. Secondly, the intervention was designed such that some recorded data were always entered by the senior resident whose role made him or her responsible for the data form during care transitions. Additionally, we agreed with physician investigators who believe it is essential to adopt a culture that encourages review of prior ED care during the care transition with the intent of identifying errors and optimizing care,^5^ and residents play a key role in any initiative to produce a culture change within an academic department.^6^ Finally, according to a study of closed malpractice claims, resident care transitions are higher risk than those not involving physicians in training.^7^

### Intervention

Incorporating published suggestions, the standardized care transition process included six features: Outgoing and incoming emergency physicians were encouraged to 1) openly discuss the care being delivered with the goal of identifying errors and optimizing patient safety and ensure that 2) care transitions were performed in a standard location, as a group, to allow the necessary information review with limited interruptions; and 3) an incoming senior resident was designated as the care transition “data resident” who was tasked with reviewing each patient’s electronic medical record (EMR) data (nursing notes, laboratory data, imaging, pending orders and vital signs). The data resident was accountable (by signature) for identifying inaccurately reported data. (All incoming residents and faculty were asked to document MCI that were identified later during the course of their shift.) 4) Both incoming and outgoing residents and faculty were encouraged to review each patient’s EMR data during the care transition to identify inaccuracies. 5) The incoming residents and faculty were given the opportunity to ask additional questions at the end of each patient-care transition report and were queried regarding comprehension of the report.(6) The outgoing faculty or most senior outgoing resident was designated the “interruption manager” and was responsible for handling ongoing ED issues to minimize interruptions of the care transition process.

### Outcome Measures

The primary outcome measure was the number of missed clinical items. We defined MCI as items not properly communicated during checkout that resulted in a delay or change in disposition or treatment plan. There were six MCI categories: 1) vital signs; 2) laboratory data; 3) radiology data; 4) ancillary data such as ECG; 5) consultant information; and 6) an “other” category for miscellaneous information such as history of present illness element or physical exam finding.

Secondary outcome measures were the mean duration of the care transition and physician satisfaction with the intervention.

### Data analysis

The completed care transition data forms were stored in a secure folder in a standard location in the department and were collected weekly by study investigators. A single study investigator performed transcription of the data forms into a data worksheet in Microsoft® Excel 2011 (Microsoft Corporation, Richmond, VA), which was used for analysis. The numbers and types of MCI, the care transition time duration, and the number of patients signed out are presented as totals and means with associated 95% confidence intervals (CI).

We developed an online survey using SurveyMonkey®. The survey items were reviewed using an iterative process of focus group feedback to validate the survey instrument. Upon completion of the post-intervention phase, we sent the survey to the ED residents and faculty who participated in the study. Their perceptions of the intervention and their satisfaction with the new process are presented as percentages.

## RESULTS

Of the 448 resident-to-resident care transitions eligible for study inclusion, 321 (72%) had data forms submitted. During the pre-intervention phase, data from 164 (74 %) of the possible 222 care transitions were captured. Post-intervention, 157 (69 %) of the eligible 226 care transitions were included.

As shown in Table 1, 94 MCI were reported during the pre-intervention phase, and 36 during the post-intervention phase. Significant reductions in MCI were noted in the laboratory, radiology and “other” categories. There were no noticeable reductions in the ancillary data or consultant information categories. The mean MCI per care transition decreased by 58% from 0.57 (95% CI [0.41–0.73]) to 0.23 (95% CI [0.11–0.35]) as shown in Table 2.

The following are examples of MCI designated as clinically relevant. In the case of laboratory values, a patient with dizziness in whom discharge was planned was noted to have a markedly elevated blood urea nitrogen concentration that was suggestive of gastrointestinal (GI) bleeding. A GI bleed was confirmed, and the patient was admitted for observation. During the patient’s course there was a significant drop in hemoglobin and a volume loss that required crystalloid replacement. An example of a relevant MCI in the “other” category was when a patient’s past medical history was found to include AIDS, which the primary resident had not noted. The discovery led to a change in the differential and workup to include pneumocystis pneumonia and led to a change in therapy.

In the “vital signs” category of MCI, the heart rate of a patient awaiting a bed rose to 150 beats per minute. She was in atrial fibrillation with rapid ventricular response (RVR). Looking back at the electronic medical record (EMR,) it was noted that the abnormal vitals had already crossed over and were available at the time of sign-out, but since this was pre-intervention, the EMR review was not performed. The elevated heart rate was noted by nursing 45 minutes after sign-out when an inpatient bed was available. The RVR was treated and controlled, but led to a delay in transport to the inpatient bed of approximately 1.5 hours. Also in boarding patients, the systematic care transition process allowed detection of the need for re-dosing of antibiotics. Pre-intervention, re-dosing of antibiotics occurred at random and sometimes not at all, which was considered a clinically relevant MCI for patients who were being admitted and treated for active infections. We noted that pre-intervention, active secondary diagnoses needing management were also sometimes discovered at random during the shift, occasionally hours after the patient had been signed out. For example, there was a diabetic patient with a non-diabetic primary problem, but in whom hyperglycemia was worsening. During the non-systematic transition of care, the hyperglycemia was not communicated or noted, which led to a delay in the administration of insulin therapy and increased ED length of stay.

During the pre-intervention phase, the care transition start and end times were documented on 132 (80%) of the data forms, and the number of patients involved in the care transition was documented on 136 (83%). The mean and standard deviation of the care transition duration during the pre-intervention phase was 15 (95% CI 13.81, 16.19) minutes while the number of patients involved in the care transition was 9.3 (95% CI 8.69, 9.91) patients. Post-intervention, the care transition duration was documented on 124 (80%) of the data forms, and the number of patients involved in the care transition was documented on 153 (97%). The care transition duration and number of patients checked out during the post-intervention phase was similar to the pre-intervention phase, 14 (95% CI 12.99, 15.01) minutes and 9.3 (95% CI 8.73, 9.87) patients respectively.

As presented in Table 3, the majority (89.5%) of residents and faculty surveyed felt the change in the care transition process had either a positive impact or no impact. Most residents (85.7%) reported the desire to continue use of a standardized checkout process after graduation. Both residents and faculty felt the intervention decreased the number of MCI.

## LIMITATIONS

Although the participants were technically blinded to the hypothesis and outcome measures, they could have inferred this information from the data collection form and pre-intervention training. This may have resulted in a Hawthorne effect in which participants altered their behavior in an attempt to decrease MCI during care transitions. As such, the number of MCI may have been lower during the study period, which could have resulted in an underestimate of the benefit of the intervention. Participants may also have failed to either recognize or record an MCI, which could have led to underreporting.

Specific details about the circumstances leading to the MCI were not always collected and may have been helpful; whether an MCI was an omission or whether the information conveyed was deemed inaccurate was not captured, nor was the justification for considering the missed information relevant.

Instituting a verification process to assess the thoroughness and accuracy of MCI reporting would have strengthened the study and could have been achieved by having trained impartial observers identify and record MCI during the care transition and subsequent shift. However, it wasn’t feasible to have study personnel present during each shift for the four-month study period.

The study also doesn’t address whether continued long-term use of the intervention in regular clinical practice results in sustained reduction of communication errors. As the standardized process is still being used, a new phase of the investigation is planned to evaluate the intervention again and determine the current rate of MCI per care transition.

## DISCUSSION

From 1993 to 2003, ED visits in the U.S. grew by 26% while the number of EDs declined by 425 and the number of hospital beds declined by 198,000.^8^ As ED length of stay increases, mortality increases.^9^ ED crowding and boarding will likely continue to rise, subjecting patients to more physician sign-outs.

The ED transition of care is well known to be an error-prone event because pertinent patient information is often not conveyed correctly or in full. The recognized need for process improvement has led to a variety of recommendations within the medical literature. Our study evaluated a standardized care-transition process that incorporated six key elements from published suggestions.^4^,^10^,^11^,^12^

In a 2011 observational study, investigators reported that certain care-transition characteristics were associated with communication errors including un-ideal location of care transition, interruptions, and lack of access to support materials including labs and images.^10^ Taking this into account, our intervention required checkout to take place in a designated area with adequate room and a sufficient number of EMR computer kiosks for the process. An interruption manager was assigned to decrease the impact of disruptions. The outgoing faculty or most-senior outgoing resident acted as interruption manager because the role required a clinician who understood the current state of the ED and possessed the knowledge to manage any acute issues.

Current literature suggests that actively using the EMR during transfer of care improves communication at care transition.^4^,^10^,^12^,^13^ A senior resident was responsible for reviewing all available EMR data and providing up-to-date patient information on demand. Not surprisingly, the biggest post-intervention decrease in MCI were in the categories found in the EMR, such as vital signs, laboratory data, and radiology data. Of note, ECG and consultant data, which were typically not available for review in the EMR at the time of care transitions, were categories that showed no post-intervention improvement. These findings suggest that clinically relevant data not available in the EMR should be gathered and reviewed during the care transition.

There was no attempt to standardize or script the verbal content of care transitions in our intervention because there was concern that complex cases might warrant a more detailed presentation than scripting would allow. However, recent studies suggest that a standardized checklist is an effective communication tool during care transitions, resulting in statistically significant improvements in accuracy and completeness of information transferred.^14^ A 2010 study evaluated a mnemonic checklist to standardize and shorten the amount of information that was transferred. The result was a decrease in checkout duration, an improvement in the perception of the quality of the process, and a decline in the amount of missing or wrong information.^12^ Incorporating a checklist is a process change that is being considered for the future.

Very few resources are needed to implement a care-transition protocol like the one we studied. At our institution, after a brief in-service, most providers were able to implement the protocol on their next shift without further instruction. Four years after the study, the intervention is still being used and no further education has been required. Since the habits formed in residency are often maintained in future practice, this type of intervention might benefit training programs. A study of academic EM training programs found that only 10.5% have a written policy regarding care transitions in their department and almost 75% of programs don’t provide formal didactic training sessions on the checkout process.^4^

Prolonging the duration of transfer of care was a concern for providers when the process change was proposed. Our data suggest that there is no difference in duration with the implementation of the standardized process at our institution. This is consistent with prior studies with care transition interventions.^12^,^14^ In addition to the benefit of having an assigned interruptions manager, the more organized care-transition structure may have increased the providers’ focus and limited unnecessary discussion.

We recognize that board-certified EPs signing out to each other in the community are not likely to have a large team in which they will have the luxury of an interruptions manager. They may, therefore, only make use of part of the systematic sign-out process. We do strongly feel that training residents to review their patient’s EMR at checkout provides a good foundation for their future practice, particularly when there are complicated patients boarding in the ED. It is very easy to overlook the re-dosing of antibiotics, the administration of insulin, venous-thromboembolism prophylaxis and other clinically relevant therapies. Our systematic care-transition process has made checking for these things a matter of routine.

## CONCLUSION

We studied 321 care transitions, accounting for the transfer of information on roughly 3,000 patients. A strength of our study was that, in contrast to many others of its type which only used subjective measures to determine outcomes, we used an objective measurement for error. We found that the intervention, a standardized checkout process with six elements, reduced the communication error rate by 58%. The new process also improved physician satisfaction without increasing the length of time spent transferring care. As a result, our department continues to use the standardized process to this day.

## Supplementary Information



## Figures and Tables

**Table 1:** Missed-clinical-item totals by category before and after the intervention implementing a standardized sign-out checklist for patient care transitions.

Missed clinical items	Pre-intervention	Post-intervention
Vital signs	7	3
Laboratory data	37	2
Radiology data	15	9
Ancillary data	6	9
Consultant information	7	7
Other	22	6
Total	94	36

**Table 2:** Mean missed clinical item (MCI) per transition before and after the intervention.

MCI per care transition	Pre-intervention	Post-intervention
Mean (95% CI)	0.57 (0.41–0.73)	0.23 (0.11–0.35)

**Table 3:** Post-intervention survey results of 17 emergency medicine faculty and 21 residents.

Questions	Responses
1. What percentage of the time is the standardized care transition protocol being used?	
<25%	12.1%
25–50%	11.7%
50–75%	30.7%
75–99%	35%
100%	2.6%
2. Has the new care transition process decreased the amount of MCI during care transition?	
Yes	73.5%
No	15.9%
3. How has the revised care transition process impacted your overall satisfaction with care transition?	
Negatively	0.0%
Unchanged	41.9%
Positively	47.6%
4. In your first job as an attending, if no formal care transition process were in place, would you either use or implement our revised care transition process? (Residents only)	
Yes	85.7%
No	14.3%
5. With the implementation of the revised care transition process, do you feel that interruptions have a smaller impact on overall care transition? (Faculty only)	
Yes	47.1%
No	52.9%

*MCI,* mild cognitive impairment
