# Supplementary material for: Improved Accuracy and Quality of Information During Emergency Department Care Transitions
Source: West J Emerg Med. 2017 Feb 27;18(3):459–65. doi: 10.5811/westjem.2016.12.30858 (PMC5391896; doi:10.5811/westjem.2016.12.30858)
Supplement: Supplementary file 1 [file wjem-18-459-s001.pdf]

Appendix 1

ED Handoff Assessment Log	Date:	Sign-out Start Time:<br>Sign-out End Time:

Senior/Data Resident:

Outgoing Staff		Incoming Staff	
Attending:		Attending:	
Resident 1		Resident 1	
Resident 2		Resident 2	
Resident 3		Resident 3	
Resident 4		Resident 4	

Was the current handoff process used? Yes No
Was the attending present for handoff? Yes No

List ALL altered/delayed dispositions or managements that occurred on this shift secondary to clinical items that were either not transferred (NT) or inaccurately transferred (IT) during the patient handoff.

#	Clinical Item	NT	IT	Altered/delayed disposition or management
1				
2				
3				
4				
5				
6				
7				
8				
9				
10				
11				
12				
13				
14				
15				
16				
17				
18				
19				
20				

List ALL items in which the checklist added relevant knowledge, changed patient treatment or changed disposition.

#	Clinical Item	#	Clinical Item
1		5	
2		6	
3		7	
4		8	
